# Paternal adaptation and related factors in Iranian fathers

**DOI:** 10.5935/1518-0557.20210044

**Published:** 2022

**Authors:** Negin Jazayeri Nezhad, Zahra Bostani Khalesi, Maryam Niknami

**Affiliations:** 1 Instructor, Department of Midwifery/ Research Center of Social Determinants of Health, University of North Khorasan Medical Sciences, Bojnourd, Iran; 2 Associate Professor, Social Determinants of Health Research Center, Guilan University of Medical Sciences, Rasht, Iran; 3 Instructor, Social Determinants of Health Research Center, Guilan University of Medical Sciences, Rasht, Iran

**Keywords:** fathers, adaptation, paternal behavior

## Abstract

**Objective:**

Although fathers are an essential element in family health, they are often overlooked by the healthcare system and are not given enough information on how to care for their infants and spouses. In this regard, understanding the characteristics or predictors of paternal adaptation might help healthcare providers design and implement interventions to foster paternal adaptation. The present study looked into paternal adaptation and related factors in Iranian fathers.

**Methods:**

This cross-sectional study included 298 fathers seen at 41 comprehensive healthcare centers in Rasht. The sampling method was stratified and each comprehensive health center was considered as a separate group. We measured paternal adaptation using the standardized paternal adaptation questionnaire (PAQ) with 38 items and five domains.

**Results:**

The results indicated that paternal adaptation scores were statistically different based on child age, length of marriage, and history of miscarriage (*p*<0.05). Father age presented a significant inverse correlation with the adaptation score, with lower paternal adaptation scores observed in older fathers (r=-0.115, *p*=0.048).

**Conclusions:**

The most significant factors related to paternal adaptation were history of miscarriage, satisfaction with family wellbeing welfare and income level, nationality of the father, maternal age, and satisfaction with married life.

## INTRODUCTION

Fatherhood is an important development in a man’s life and causes dramatic changes in one’s personality, lifestyle, identity, and development (Barenski, 2010). Fatherhood is a process that causes evolvement in a man and has different dimensions that affect the family, parents, and even society (Doherty *et al*., 1998).

The changes introduced by fatherhood lead to the formation of a new identity that shifts as reality evolves over time, leading to differences in the personal-social relationships of men who became parents (Condon *et al*., 2004). This requires constant effort so that men can adapt to fatherhood in the personal and social dimensions (Katz-Wise *et al*., 2010).

In recent years, many researchers have considered the notion of adaptation, which revolves around the efforts made by families to adjust to the arrival of children while addressing personal and environmental needs (Bowen, 1990). Paternal adaptation involves the efforts made by men as they take on their roles as fathers along with the related responsibilities and challenges (Ricci & Kyle, 2009).

Studies have reported underlying factors such as economic and social status, income level, and education levels (Herrick *et al*., 2004), in addition to smoking, drinking alcohol, and substance abuse, marital satisfaction, and planning for parenting as predictors of paternal adaptation (Yanes *et al.*, 2017). Feinberg & Kan (2008) argued that the spouse’s age did not affect paternal adaptation. Other authors found that paternal ethnicity and race did not affect paternal adaptation or performance (Katz-Wise *et al*., 2010), while another study indicated an effect of race and ethnicity on paternal performance. For instance, the results of a study by Hijjawi *et al*. (2003) indicated that factors affecting paternal participation and adaptation included living with the spouse and children, although other factors such as drinking alcohol, smoking, and adherence to cultural principles, education, and income levels also affected the performance of African-American fathers. In this regard, the study of Glauber & Gozjolko (2011) also showed that paternal adaptation is influenced by race, ethnicity, and culture.

Health centers rarely provide any training for men, with most of the training being directed to women (Bardige & Bardige, 2008). In general, men are not considered as the recipients of health services, an issue that causes many problems for them as they become fathers. In addition to not receiving the necessary training to cope with their responsibilities as parents, fathers are not aware of what a paternal role is or the factors that may greatly affect their adaptation to their paternal roles (Doherty *et al*., 1998). Studies have provided contradictory results about the effects of these factors on paternal adaptation. Therefore, recognizing fatherhood and adapting to a paternal role requires extensive information in different countries and cultures (Alessia & Roufeil, 2012).

Given the importance of the father’s role in the family, a figure that affects all aspects of a child’s life and society, and considering the conflicting results about the factors described in studies to determine how successfully fathers adapt to their new roles, and the low number of studies in Iran about paternal adaptation scores and related factors, the present study aimed to look into paternal adaptation and related factors in Iranian fathers.

## MATERIAL AND METHODS

**Figure 1:**
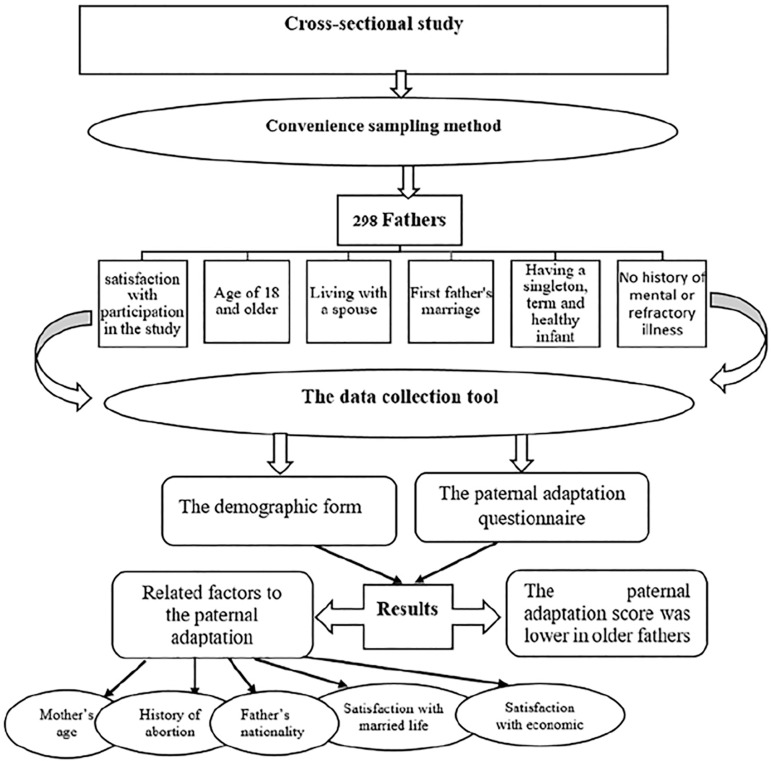
Schematic diagram of the research process.

This cross-sectional study enrolled 298 fathers seen at a comprehensive health centers in Rasht arranged in a convenience sample after meeting predefined inclusion criteria (Figure 1).

The inclusion criteria were as follows: willingness to join the study; literacy; age of 18 years or older; no history of mental or refractory illness; living with a spouse; fathers on the first marriage; mothers on the first marriage; first experience of fatherhood; and having a full-term healthy single child (aged one month to one year). The exclusion criterion included reluctance to continue participating in the study.

Data collection included a questionnaire consisting of two sections. The first section was a demographic form designed by the investigators to determine predictors of paternal adaptation and examine factors cited in previous studies. This section assessed factors relating to the father’s personal-social characteristics (age, level of education, ethnicity, occupation, average monthly income, alcohol and drug use, smoking, and hookah use); the spouse’s personal-social characteristics (age, ethnicity, education level, occupation, and average monthly income); the child’s personal characteristics (age, sex, whether the parents were satisfied with the child’s gender, and health status); and pregnancy history (planning for parenthood, history of infertility in the mother, history of miscarriage, and fetal death).

The second section was a standardized tool to assess paternal adaptation, in which the total Content Validity Ratio (CVR) was 68% and the total Content Validity Index (CVI) was 92%. The internal reliability of the tool was 89% based on Cronbach’s alpha test, and the external test-retest reliability was 96% (Eskandari *et al*., 2018a). The paternal adaptation questionnaire (PAQ) had 38 items and five subscales: ability in performing the roles and responsibilities (13 items); perceiving parental development (8 items); stabilization in paternal position (8 items); spiritual stability and internal satisfaction (6 items); and challenges and concerns (3 items). The items in the questionnaire were rated based on a five-point Likert-type, with scores of 5 (always/strongly disagree), 4 (often/agree) 3 (sometimes/neutral), 2 (rarely/disagree), and 1 (never/strongly disagree). The Ethics Review Committee at the Gilan University of Medical Sciences approved this study and granted it certificate IR.GUMS.REC.1396.474.

The author introduced himself, stated the purpose of the study to the participants, and informed them of the voluntary nature of their participation in the study and the confidentiality with which their information would be treated before obtaining their written consent to join the study.

The data collected from the participants were entered into SPSS 21. To calculate paternal adaptation scores and separate the findings into questionnaire domains, the author used statistical indices including mean, median, minimum, maximum, and standard deviation. The author used the independent t-test and ANOVA to compare paternal adaptation scores according to demographic variables. Pearson’s correlation coefficient was used to investigate the correlations between scores and quantitative variables. When the hypotheses of the parametric tests above were not true, the author used similar nonparametric tests, namely Wilcoxon, Kruskal-Wallis, and Spearman’s rank correlation coefficient. To determine predictors of paternal adaptation, the author utilized multiple linear or logistic regression models; statistical significance was set at 5%.

## RESULTS

The mean age of the fathers included in the study was 94±4 years; 87.9% had Gilani ethnicity; 44.6% had an academic education; 53.4% were self-employed; 87.9% drank alcohol; 94.6% used drugs; 83.9% used a hookah; and 94% did not smoke. The mothers’ mean age was 24±4 years; 49.3% of the mothers had an academic education; 87.6% were Gilani; 82.9% were housewives; and the monthly income of 84.6% of working mothers ranged between 10 and 20 million rials. The children’s mean age was 3.34±3.57 months; 55.3% were boys and 94.3% of fathers were satisfied with their children’s gender. More than half (52.7%) of the fathers were satisfied with their economic welfare status; 85.2% participated in child care; 86.9% lived with their spouses and children; 49% had an income between 10 and 20 million rials; 94% were satisfied with living with their spouses. The average number of visits of fathers to comprehensive health centers was 2.15±1. The mean length of marriage was 4.35±2.57 years.

The distribution of paternal adaptation scores and subscales are presented in Table 1. There was a significant inverse correlation between the age of the father and the adaptation score, meaning that older fathers had lower paternal adaptation scores (r=-0.115, *p*=0.048). It should be noted that the correlation with maternal age was also inverse in the table, but it was not statistically significant (*p*=0.069).

**Table 1:** Distribution of Paternal adaptation and its subscales.

Maternal - Infant Relationship	M (SD)	CI	
		Lower limit	Upper limit
Ability to perform the roles and responsibilities of becoming a father	95.42 (7.11)	94.61	96.23
Ability to perceive the parental development	91.59 (10.48)	90.40	92.78
Stabilization in paternal position	89.41 (12.27)	88.01	90.81
Spiritual stability and internal satisfaction	95.32 (8.48)	94.35	96.28
Challenges and concerns	35.88 (27.44)	32.75	39.01
Paternal adaptation	88.63 (6.62)	87.88	89.39

Paternal adaptation scores were statistically significant related to length of marriage (*p*=0.035) and history of miscarriage (*p*=0.006), with higher paternal adaptation scores seen in families with a length of marriage of 2-5 years; and families with a history of miscarriage had lower paternal adaptation scores.

The paternal adaptation score was statistically related to the child’s age, with higher scores found in parents with children aged 1-to-2-months (*p*=0.028).

A multiple linear regression model was used to find predictors of paternal adaptation based on the personal and social variables of the mothers, fathers, and children. To increase the efficiency of the model, all variables with significance levels of less than 0.25 in univariate analyses on Table 2 were included in the model. We used a stepwise multiple linear regression method. The fit of the model was confirmed based on the results of analysis of variance (ANOVA) (*p*<0.001).

**Table 2:** Distribution of Paternal adaptation and its subscales.

Variables	Total scores	
Father's age	r	-0.115
	p	0.048
Mother's age	r	-0.105
	p	0.069
Length of marriage	r	-0.039
	p	0.504
Child’s age	r	-0.037
	p	0.525
Number of times fathers went to the health centers	r	-0.037
	p	0.527
Fathers’ participation in health cares	r	-0.044
	p	0.453

*p*: Significant level r: Spearman's rank correlation coefficient

Table 3 presents the results of the multiple linear regression model used to predict paternal adaptation. According to the model, the most important predictors of paternal adaptation were history of miscarriage *(p*=0.006), satisfaction with the economic welfare status (*p*=0.026), father’s ethnicity (Guilanian *vs*. non-Guilanian) (*p*=0.021), the mother’s age (*p*=0.034), and satisfaction with living with the spouse (*p*=0.043). Families with a history of miscarriage had lower levels of paternal adaptation. Individuals with higher levels of satisfaction with the economic status (*p*=1.6) and satisfaction with living with the spouse (B=2.59) had higher paternal adaptation scores, while increasing maternal age (B=0.17) decreased paternal adaptation scores.

**Table 3:** Regression coefficients of the paternal adaptation determinants based on the multiple linear regression model.

Model	CI		*p*	SD	R
	Maximum	Minimum			
Fixed amount	89.840	88.246	0.000	0.405	89.043
History of miscarriage	-1.058	-5.583	0.004	1.150	-3.321
Fixed amount	89.242	87.043	0.000	0.559	88.143
History of miscarriage	-1.259	-5.762	0.002	1.144	-3.510
Satisfaction with economic-welfare status	3.240	0.266	0.021	0.756	1.753
Fixed amount	87.945	79.347	0.000	2.184	83.646
History of miscarriage	-1.233	-5.710	0.002	1.137	-3.471
Satisfaction with economic-welfare status	3.151	0.19	0.027	0.752	1.670
	4.654	0.182	0.034	1.136	2.418
Fixed amount	93.995	82.018	0.000	3.043	88.007
History of miscarriage	-1.176	-5.631	0.003	1.132	-3.404
Satisfaction with economic-welfare status	3.156	0.211	0.025	0.748	1.683
Father’s ethnicity	4.792	0.336	0.024	1.132	2.564
Mothers age	-0.006	-0.323	0.042	0.080	-0.165
Fixed amount	90.702	75.381	0.000	3.892	83.042
History of miscarriage	-0.896	-5.360	0.006	1.134	-3.128
Satisfaction with economic status	3.132	0.202	0.026	0.744	1.667
Father's ethnicity	4.839	0.405	0.021	1.126	2.622
Mother's age	-0.013	-0.328	0.034	0.080	-0.170
Satisfaction living with a spouse	5.095	0.077	0.043	1.275	2.586

CI: Confidence Interval

*P*-value: P

SD: Standard deviation

R: Regression coefficient

## DISCUSSION

The findings of the present study revealed an inverse relationship between fathers’ age and paternal adaptation scores, in which older fathers had lower paternal adaptation scores. The results of Eskandari *et al*. (2018b) also indicated that the father’s age solely correlated with the perception of paternal evolution, but multivariate regression did not confirm this relationship. However, the findings of a study by Mortazavi & Keramat (2012) showed that there is a direct relationship between a father’s age and adaptation to the paternal role. The study by Sevil & Ozkan (2009) also showed that the father’s age is strongly related to on paternal adaptation, with older fathers presenting higher levels of adaptation to the paternal role. 

To justify the contrast between the results of the present and other studies, it should be noted that our study enrolled fewer individuals than other studies, which caused father’s age not to be one of the factors affecting paternal adaptation. 

The results of the present study revealed an indirect and significant relationship between the age of the mother (spouse) and paternal adaptation scores, in which scores decreased as ages increased, a finding in line with the study by Madhavan *et al*. (2014). Nonetheless, Eskandari *et al*. (2019) reported that a spouse’s age did not affect paternal adaptation scores or its dimensions. Cultural and ideological differences observed between the authors of this study and of the study published by Eskandari (conducted in Qom City) may explain this discrepancy. As a Guilan mother get older, the father of their children feels he can more confident entrust the mother with more responsibility in caring for their children, which may in turn affect the father’s adaptation to the paternal role.

The results of the present study did not find a significant relationship between child age and paternal adaptation scores, as described by Carneiro *et al*. (2012). However, the findings of a study by Benjamin *et al.* indicated that as the child’s age increases, the father-child bond becomes stronger, thus producing increased feelings affection, warmth, and support (Goodman *et al*., 2008). Many fathers stated that as their children became older, they too became more confident about their parenting skills (Cabrera *et al.,* 2011). 

The reasons for this contradiction might be justified by the increased need for care and attention neonates and infants require, which pushes fathers to adapt more intensely to a paternal role with the birth of a child. The individuals included in the present study had children aged from one month to one year. No significant relationship was observed between child sex and paternal adaptation scores and its domains, as also described by Eskandari *et al*. (2019).

This study found a significant relationship between paternal adaptation and child age, with highest scores associated with children of lower ages (1-2 months). In the study by Alayi *et al.*, an inverse significant relationship was observed between child age and a father’s abilities to engage in parental roles and responsibilities; as child age increased, fathers had less need to engage in paternal roles and responsibilities, which is consistent with the present study (Alayi *et al*., 2011).

## CONCLUSION

Higher scores were seen in the responsibilities, perceiving parental development, stabilization in paternal position, spiritual stability and internal satisfaction subscales, whereas lower scores were observed in the challenges and concerns subscale. 

Based on the results of the study, the factors more strongly related to paternal adaptation were history of miscarriage, satisfaction with economic welfare condition, nationality of the father, maternal age, and satisfaction with married life.

One of the limitations of this study is that its findings cannot be generalized, since the individuals included in this study were from the city of Rasht and paternal adaptation is context-specific and may vary within one same society and from city to city.
